# Activation of microglia bolsters synapse formation

**DOI:** 10.3389/fncel.2014.00153

**Published:** 2014-06-02

**Authors:** Gonçalo Cristovão, Maria J. Pinto, Rodrigo A. Cunha, Ramiro D. Almeida, Catarina A. Gomes

**Affiliations:** ^1^CNC—Center for Neuroscience and Cell Biology, University of CoimbraCoimbra, Portugal; ^2^PhD Programme in Experimental Biology and Biomedicine (PDBEB), Center for Neuroscience and Cell Biology, University of CoimbraCoimbra, Portugal; ^3^Faculty of Medicine, Biochemistry, University of CoimbraCoimbra, Portugal; ^4^Faculty of Medicine, Pharmacology and Experimental Therapeutics, University of CoimbraCoimbra, Portugal

**Keywords:** brain wiring, microglia, neurodevelopment, neuroinflammation, pre-synaptic differentiation, synapse formation

Microglial cells in the central nervous system (CNS) are major players of innate immunity, the first line of defense in the presence of danger signals, from bacterial infections to mediators released by neurons upon cytotoxic insult (Kettenmann et al., 2011). Particular attention has been paid to the involvement of microglia in pathological conditions and their eventual dual role, as disease amplifiers and/or executors of brain repair, is still a matter of controversy (Benarroch, 2013); the fact that inflammation is itself a strategy to contain biological threats, which inevitably leads to variable degrees of damage, may explain the apparent duality of roles attributed to microglia. These microglial functions are mainly dependent on the release of a plethora of mediators (from trophic factors to anti- and pro-inflammatory cytokines or chemokines) and are accompanied by characteristic changes of morphology, ranging from highly ramified to amoeboid shapes (Kettenmann et al., 2011).

Although the role of microglia has been mostly approached from the angle of pathology, microglial cells are also active players in healthy conditions by constantly monitoring the brain parenchyma (Davalos et al., 2005; Nimmerjahn et al., 2005) and correcting deviations from homeostasis; accordingly, several studies reported that the genetic alteration of molecules selectively blunting specific microglial functions impacted on synaptic transmission and synaptic plasticity (Pascual et al., 2012; Ji et al., 2013; Zhang et al., 2014), leading to deficits of behavioral response, such as social interaction, motor learning, or short-term memory (Rogers et al., 2011; Parkhurst et al., 2013; Zhan et al., 2014). These functional effects of microglia on synaptic plasticity and the direct observation of physical apposition of microglia processes with synapses led to the emergence of the concept that microglia could directly interact with synapses (Wake et al., 2009; Tremblay et al., 2010), forming the so called quad-partite synapses (Schafer et al., 2013). This was re-enforced by the observed ability of microglia to engulf and remove synapses, a process named synaptic pruning, which is crucial both for adequate synaptic wiring during neurodevelopment, as well as during synaptic re-wiring following brain injury (Wake et al., 2009; Schafer et al., 2012; Bialas and Stevens, 2013; reviewed in Kettenmann et al., 2013).

The recent report of Chugh et al. (2013) provides evidence supporting a more complex role for microglia-associated neuroinflammation in the process of synaptic wiring. In fact, this report shows that the creation of an inflammatory environment in the hippocampus of adult mice triggers a region-selective increase in the number of thin dentritic spines endowed with PSD-95, indicative of enhanced synaptic connectivity of newborn neurons, without overt changes in neuronal or astrocytic morphology. This is in striking agreement with other reports showing that the functional impairment of specific microglia functions or the ablation of microglia triggered an increase of excitatory synaptic transmission (Pascual et al., 2012; Ji et al., 2013).

However, it remained to be shown if this ability of microglia to bolster synapse formation resulted from a direct signaling of microglia onto maturating synapses and there is still no information about the putative ability of microglia to control the axonal sprouting. This led us to design an experimental protocol to study the direct interaction between microglia and immature axon/pre-synaptic terminals. This was achieved using microfluidic chambers where hippocampal neurons are plated in one compartment and, as the neurons develop, the axons grow through the microgrooves to reach a second physically isolated compartment (Taylor et al., 2005; Neto et al., 2014). The growing axons, but not the cell bodies, were exposed to the N9 microglia cell line, which was previously *primed* with lipopolysaccharide (LPS), a “classic” microglia activator. When we measured the density of nerve terminals by immunofluorescence against the pre-synaptic marker synapsin I, we found that activated microglia caused a clear-cut increase in the axonal density of the protein, which was not observed in the presence on non-*primed* microglia or in isolated axons (Figure 1). Recently, Parkhurst et al. (2013) demonstrated that microglia regulates synapse formation through brain-derived neurotrophic factor (BDNF), which is in line with our previous work (Gomes et al., 2013), showing the ability of LPS to increase BDNF release by N9 microglial cells. The increase in the number of synapsin clusters is, however, more striking in axons which are in contact with microglial cells, suggesting that a cell adhesion molecule might also mediate this effect (inset, Figure 1). Further studies using conditioned medium from LPS-*primed* microglial cells may help clarify the relative contribution of diffusible mediators (such as BDNF) and axon-microglia physical contact in the ability of *primed* microglia to bolster synapse formation.

**Figure 1:**
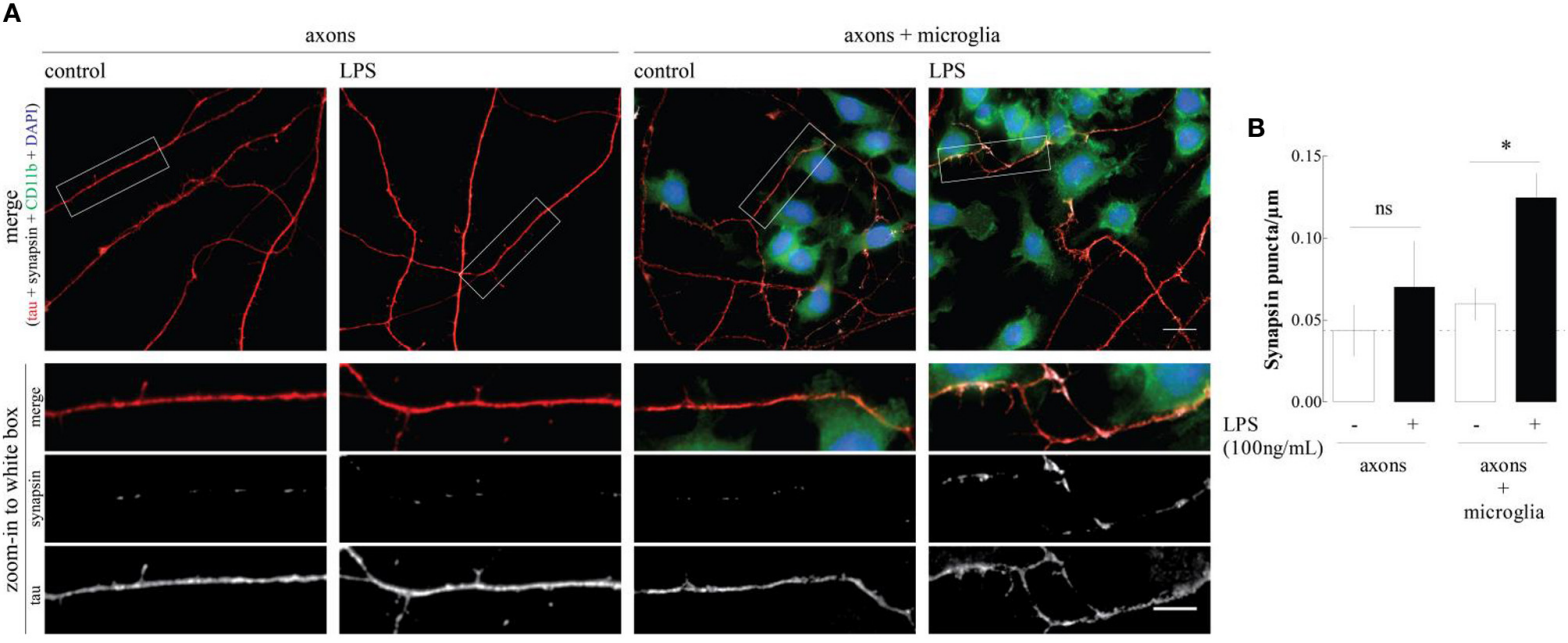
**LPS-*primed* microgial cells increase synapsin puncta along axons. (A)** Representative images of the axonal/microglia compartment of microfluidic chambers in which rat embryo hippocampal neurons were allowed to grow for 7 days. When indicated, before axons arrival to the axonal compartment a microglia cell line was cultured (DIV3) and activated with LPS for 6 h (DIV4). Cultures were stained for CD11b (microglia marker, green), DAPI (nuclei, blue), tau (axonal marker, red), and synapsin I (synaptic vesicles marker, white). Scale bars are 10 and 5 μm for upper and lower zoom-in images, respectively. **(B)** Quantification of the number of synapsin puncta per axonal length. Results, obtained analyzing 20 images per condition, are expressed as mean ± s.e.m. of three independent experiments, (^*^p < 0.5, by Two-Way ANOVA followed by Bonferroni *post-hoc* test).

Overall, these finding show that microglia are more than synaptic strippers, but are actually important controllers of synapse formation. This novel role of microglia on pre-synaptic differentiation may be of particular importance to better understand neurodevelopment disorders characterized by aberrant synapse formation associated with maternal infections during pregnancy.

## Conflict of interest statement

The authors declare that the research was conducted in the absence of any commercial or financial relationships that could be construed as a potential conflict of interest.
